# Community perceptions of mental illness in rural Uganda: An analysis of existing challenges facing the Bwindi Mental Health Programme

**DOI:** 10.4102/phcfm.v9i1.1404

**Published:** 2017-10-11

**Authors:** Arya Shah, Lydia Wheeler, Kristen Sessions, Yusufu Kuule, Edwin Agaba, Stephen P. Merry

**Affiliations:** 1 Department of Family Medicine, Mayo Clinic College of Medicine, United States; 2 Departments of Community Health, Batwa, and Mental Health, Bwindi Community Hospital, Uganda

## Abstract

**Objectives:**

To assess community perceptions of mental illness in the Bwindi Community Hospital (BCH) catchment area: to recognise beliefs about the causes and the treatments for mental illness. To provide community data to staff at BCH as they work to develop more effective community mental health programmes.

**Background:**

A shortage of mental health providers in Uganda has prompted research into community-based task-sharing models for the provision of mental health services in underserved communities.

**Methods:**

Six focus group discussions, with a total of 54 community members (50% male, *n* = 27; mean age + s.d. [39.9 + 10.9 years]) from the BCH catchment area, were conducted to assess community member and stakeholder perceptions of mental illness and belief in the feasibility of community-based programming. Qualitative study of data through thematic analysis was conducted to assess the presence of commonly occurring perceptions.

**Results:**

Qualitative thematic analysis revealed two major themes: (1) belief that any given patient’s metal illness results from either an intrinsic or an extrinsic cause and (2) belief in a need to determine treatment of mental illness based on the believed cause.

**Conclusion:**

As BCH designs community-based mental health services, our findings provide support for the need for further education of community members and training of community health workers to address and integrate the above-stated beliefs regarding mental illness.

## Introduction

### Background

Significant treatment gaps in the realm of international mental health^1^ have brought mental health to global attention. Programmes such as Emerging Mental Health Systems in Low- and Middle-Income Countries (EMERALD) and Programme for Improving Mental Health Care (PRIME) aim to create sustainable mental health solutions in low- and middle-income countries (LMIC). Studies have cited the utility of a task-sharing, community-based model as a means of providing mental health services for a variety of mental health disorders to underserved populations.^2,3^

Understanding existing community perceptions of mental health is vital to establishing successful practices.^4^ The UK Department of Health looked at stakeholder perceptions in Ethiopia, India, Nepal, South Africa, and Uganda regarding the feasibility of a task-sharing model for the treatment of mental health. Results showed that community members, politicians, health workers, and leaders acknowledged the benefits of a task-sharing model to increase access to services but also identified several challenges that would need to be addressed in implementing such a model; these challenges included a lack of knowledge among providers about identifying mental illness and deeply rooted stigma surrounding mental disorders and their treatment.^5,6,7^

The above studies outline broad themes and challenges across several countries as they relate to the creation of community-based mental health programmes in LMICs. However, it is vital to address these challenges in community-specific contexts when creating community health programmes.^8,9^ Studies have shown that satisfaction with treatment programmes is correlated with interventions developed within communities or adapted to specific community needs.^3,6,7^ Therefore, in-depth analysis is needed to identify the unique barriers to the establishment of effective mental health programmes in a given community.^10,11,12^

The present study was conducted in partnership with the Bwindi Community Hospital’s (BCH) Mental Health Programme in south-western Uganda to examine the knowledge, attitudes, and beliefs about causes, manifestations, and treatment of mental illness among community members and key stakeholders. The goal of our quality improvement project was to determine the acceptability and feasibility of establishing a community-based mental health programme in the BCH catchment area. The study (1) identifies existing beliefs that currently impeded the development of effective community-based mental health programmes and (2) provides insight of community stakeholders on the challenges to establishing effective community-based care.

Our project was coordinated with BCH staff, who wished to improve the quality of their existing mental health service programme and therefore asked us to assist them in gathering information on community and health sector perceptions for planning the subsequent steps in initiating such a project. BCH has its own quality improvement protocol and our research team was provided approval through this entity. Our study was deemed Institutional Review Board (IRB) exempt by the Mayo Clinic Institutional Review Board and the leadership and staff of Bwindi Community Hospital. Our study was deemed IRB exempt based on the standard language utilised by IRBs worldwide, including the Makerere and Mbarara official Ugandan IRBs and UNCST accredited Research Ethics Committees.

### Setting

The BCH catchment area (00º45’ 03.1’’ S, 29º42’ 03.6’’E) is in the District of Kanungu in south-western Uganda. The district has an estimated population of 252 100.^13^ The Kanungu district is remote with limited infrastructure and service delivery.^13,14^ Eighty per cent of the population lives in rural settlements.^15^ The majority of the people are of Bakiga ethnicity, while the remainder (~900 individuals) are the indigenous Batwa population who were displaced with the 1993 Bwindi National Park designation.^14,16^

BCH is a non-governmental 112-bed hospital founded in 2003. It is staffed by 121 personnel including doctors, nurses, midwives, health workers, and support staff. Seventy per cent of the staff are from the BCH catchment area, while the remaining 30% are from other areas of Uganda with frequent volunteer physicians from the United Kingdom. Of the 10 physicians, five generalists and one obstetrics/gynaecology practitioner provide mental health treatment as part of their outpatient practice. A formal mental health programme, staffed by a hospital-based psychologist and mental health nurse and approximately 30 community health workers, was initiated here in 2013.^16^ Currently, the programme provides care for community members suffering from mental illnesses such as psychosis, depression, and anxiety in addition to substance addiction services and epilepsy treatment.

### Current state of mental healthcare in Uganda

A 2007 study previously reported that 35% (*n* = 9.6 million, based on Ugandan population at that time) of Uganda’s population currently suffers from mental illness; while just under half of these individuals require some form of treatment for their illness,^17^ most never seek mental health services.^18^ Since 2007, the World Bank reports that the population of Uganda has increased from approximately 30 million to over 39 million.^19^ It is likely that the prevalence of mental illness continues to increase as the population continues to grow.

Stigma surrounding mental health and its treatment is one of the greatest barriers to mental healthcare. Despite the high prevalence of mental illness in Uganda, previous studies have consistently demonstrated the presence of stigma not only among the general population but also among providers.^20,21^ HIV patients, survivors of abuse, and rape survivors are at increased risk but often fail to seek treatment because of stigma and fear of retribution.^22^ There are 28 inpatient psychiatric units throughout Uganda, and only one mental hospital. Over 60% of all available beds are located either within or in close proximity to Kampala, the largest city in Uganda.^23^ However, previous census results have shown that 87.7% of the population lives in rural areas.^24,25^ Such a maldistribution of resources bolsters the argument for community-based mental healthcare. Recent efforts to integrate mental health management into the system of primary healthcare in Uganda are yet to be fully realised in rural communities; such efforts to decentralise care have acknowledged the role that community perceptions and stakeholders play in the realisation of mental healthcare models.^26,27^ The present study aims to provide BCH with concrete data on community stakeholder perceptions of mental illness to educate providers and decentralise mental healthcare services.

## Methods

### Participants

Participants were identified in two ways. We sought to achieve proportion sampling representation across adults and communities as identified by partners from BCH. Six communities were chosen to allow for this: Kyumbugusho, Nkwenda, Kanyamasinga, Kanyashande, Mukono, and Buhoma (Table 1). This allowed for contextual variation, representation of the population, and theme saturation as verified through the data analysis process. Forty-two households (seven from each community) were chosen using a probability sampling method from a list of primary sampling units based on geographic clusters.^15,28^ In this way, we initially obtained a set of randomised initial informants that were as diverse as possible in our probability proportional sampling (which required *n* = 45 for sufficient sampling). The purpose of the study was discussed with the adults of the household and consent to participate in the focus group was sought. Of the 36 adults who were approached, 33 (91.7%) consented. Our primary focus was on quality improvement and potential implementation and acceptance of a BCH mental health programme. Thus, we wanted to make sure that (1) patients with mental illnesses and (2) community leaders who have disproportionately high influence in helping families decide the treatment course of community members were sufficiently represented. While these two populations are not ‘hidden populations’ per se, we found that the sensitivity of mental illnesses and the randomisation of our initial selection did not adequately give us these populations in population proportions (35%). To this end, a snowball sampling technique^29^ was also used. Individuals were recommended by other participants based on their perceived roles in developing a community mental health programme. Of the 25 adults recommended, 21 (84%) consented (Table 2). Thus, we combined both the randomised proportional sample and the snowball sampling to make sure we would get an adequate sample of persons (*n* = 54, total participants) with mental illnesses and leaders, two groups who have disproportionate investment in determining care practices.

**TABLE 1:** Focus group participant profiles.

Community	Community members*^a^*	Service users and caregivers	Community health workers*^b^*	Total*^c^*
Buhoma	7 (1)	1	0	8 (4f: 4m)
Kanyamisinga	6 (1)	1	1	8 (4f: 4m)
Kanyashande	7 (2)	4	1	12 (5f: 7m)
Kyumbugusho	8 (0)	1	0	9 (5f: 4m)
Mukono	6 (1)	2	1	9 (5f: 4m)
Nkwenda	3 (2)	3	2	8 (4f: 4m)
**Total**	**37 (7)**	**12**	**5**	**54**

a , Values in parentheses indicate the number of community leaders. Community member occupations ranged widely from pastors, motorbike operators, gorilla protection, security guards, unemployed, farmers, janitorial and teachers.

b , The term ‘community health workers’ (CHWs) includes those working at the community level, including those not titled ‘CHWs’ such as Village Health Workers.

c , The gender of participants with ‘f’ for female and ‘m’ for male is listed in parenthesis.

**TABLE 2:** Participant demographics.

Demographics	Number of participants *n* (%)
**Sex**	
Male	27 (50.0%)
Female	27 (50.0%)
**Age**	
18–25 years old	9 (16.7%)
26–50 years old	33 (61.1%)
Over 50 years old	12 (22.2%)
**Marital status**	
Single	12 (22.2%)
Married	28 (51.9%)
Other	14 (25.9%)
**Education level**	
No formal education	14 (25.9%)
Some primary	28 (51.9%)
Primary or beyond	12 (22.2%)

*n*, number.

Following consent, participants were asked to provide responses to a series of questions regarding mental illness; these questions were outlined in a pre-formed discussion guide created by the study team based on the specific interests of BCH staff (Appendix 1). Data in each of the six sites were collected from community members (including community leaders and lay people), community health workers, service users, caregivers, and family members.

### Data collection

Data collection took place between May and August 2015 and was coordinated from BCH, Kanungu, Uganda. Because of the scarcity of research in this area and in order to address the specific questions posed by the staff at BCH, we chose a qualitative approach to obtain a baseline survey of community members’ knowledge, attitudes, and beliefs about causes, manifestations, and treatment of mental illness.^30^ The primary language in the Bwindi area is Rukiga, and all focus group discussions were conducted in Rukiga. The moderator (fourth author) is fluent in both Rukiga and English. Each focus group session took place in a private conference room and lasted between 60 and 90 min. No one was present during data collection besides the authors and focus group participants. The moderator and interviewer followed a discussion guide developed jointly by the authors and pilot-tested in the community to direct the conversation (Supplementary Material). Interviewers were free to probe further following responses to specific questions if they felt further enquiry could yield new information on the question at hand. Participants were provided with 10 000 UgSh (3.50 USD) at the end of the session. Such compensation is standard procedure for all focus groups conducted by BCH. The amount of compensation was predetermined by BCH staff.

### Data analysis

Interview data were audiotaped, de-identified and transcribed verbatim in Rukiga, translated to English, and back-translated by three bilingual Rukiga–English interpreters, whose areas of research expertise are psychiatry and community health. This was back-translated and approved by all three translators to ensure that contextualised meaning was preserved. Data were managed with Nvivo 10 software. Data were content analysed according to Tesch^31^ and Maykut and Morehouse.^32^ All transcripts were read over once initially in order to provide a general idea of the tone and scope of the information. The verbatim transcripts were coded using inductive thematic analysis^33^ to analyse the data. Each recording was coded separately by at least three authors who independently came up with labels to attach to transcribed portions that appeared to indicate important mental health perspectives. The team then came together to compare codes and revise them in an iterative fashion. Emerging overarching themes were compared with the original transcripts, and further refined, merged, and subcoded. To minimise loss of meaning and omission of important issues in the comparative analysis, we validated all findings with bilingual Rukiga–English interpreters.

## Results

### Beliefs on causes and treatment of mental illness

A qualitative thematic analysis of responses to the discussion questions among each of the six focus groups was conducted, and two major themes emerged: (1) belief that any given patient’s mental illness has either an intrinsic or an extrinsic cause and (2) belief in a need to determine treatment of mental illness based on believed cause (Table 3). Extrinsic causes of mental illness were defined as those forces that act externally on the human body and mind. In contrast, intrinsic causes of mental illness were defined as those internal choices and personal weaknesses perceived as being damaging to the mind. Examples of perceived extrinsic and intrinsic causes of mental illness are described below.

**TABLE 3a:** Emergent themes from qualitative analysis of focus group data.

Cause	Cited examples
**Extrinsic causes of mental illness**	
Drugs	Marijuana Alcohol
Infectious disease	Yellow fever HIV Epilepsy
Spiritual causes	God Satanic powers Curses Bewitchment Bad airs
Socioeconomic causes	Poverty Familial disharmony
**Intrinsic causes of mental illness**	
Flaws in an individual	Weakness of constitution Negative impact of individual’s choices

**TABLE 3b:** Emergent themes from qualitative analysis of focus group data. Proposed treatment of mental illness.

Intervention	Treatment
Medical intervention	Hospitalisation Medications Utilisation of healthcare workers
Non-medical intervention	Prayer Traditional healers Resolution of family or personal conflicts

### Extrinsic causes of mental illness

Drugs, infectious disease and seizures were all believed to be common extrinsic causes of mental illness.

‘Alcohol taking, smoking, taking drugs like marijuana, I think that can cause mental illness.’ (Male, Kanyashande, Farmer)

‘I also think that when mosquitoes bite you, you can get mental problems, especially when you get too much fever. This can disturb the mind.’ (Female, Kanyamisinga, Farmer)

‘Everyone can suffer from mental illness because even malaria which is not attended to earlier can cause a lot of disturbance when one may start over talking and people, community may take that as mental illness.’ (Male, Kanyashande, Pastor)

‘There is also drinking too much alcohol and it causes confusion in the brain and this causes mental illness.’ (Female, Buhoma, Farmor)

‘There are times when you get illness from the community and get like headache or malaria. You at times do not bother to go to the hospital for treatment and when this problem escalates, and the malaria gets too much, it can also leave you mentally unstable and unwell.’ (Female, Kyumugosho, Gorilla Keeper)

‘There are times when mental illness is brought about by for instance epilepsy. When the child is over-disturbed by this disease/problem, the head gets affected in the long run as well.’ (Female, Mukono, Teacher)

External spiritual and emotional forces were also believed to be equally potent in negatively influencing mental health. Spirits, satanic powers, bad airs, poor choices, curses, bewitchment and stars were perceived as common external causes of mental illness.

‘We cannot blame patients/victims of mental illness for this disease because they do not invite it, only that it’s an enemy that had invaded them.’ (Male, Nkwenda, Farmer)

‘Sometimes when children go to school to study, when a student become very bright, his/her friends can bewitch him/her causing mental illness.’ (Female, Kanyamasinga, Community Member)

‘No, not anyone can get mental illness because like we said, this problem can affect those persons who are drug addicts, then others whose parents/relatives invite satanic powers/demons to come and disorganise them, which ends up making their children get mental illness.’ (Female, Nkwenda, Community Member, Farmer)

‘Also, some families are more God fearing than others and these ones have fewer chances of getting mental illness where as those families that usually invite satanic powers in most things that they do, have higher chances of getting this problem. So, not all people can get mental illness.’ (Male, Kyumbugosho, Storekeeper)

In addition to satanic forces, God was also described as having the power to inflict mental illness upon those who have acted wrongly.

‘Some people who may have in the past done odd things, such as murdering fellow man, end up being paid back and punished by God for those deeds. It’s very true from what my colleagues have said, I think that for a head to get disorders results from your actions deeds, if they were bad, God has to punish you accordingly, hence making your head ill. That can make your head unstable.’ (Male, Nkwenda, Spiritual Leader)

‘Some families still possess and believe in satanic powers, they give food, drinks, perform rituals to Satan and this may make a person mad at a time. They are not well done because the gods are not pleased and thus they communicate by making some people mad.’ (Male, Kyumbugosho, Transportation Provider)

Poverty and family disharmony causing excessive amounts of interpersonal conflict and a sense of failure are stated as being contributing factors in the development of mental illness, such as in the following accounts by participants:

‘When someone loses hope after doing a lot of things and there is no success at all, it brings many thoughts and then this can cause mental illness and the person may remain mentally ill.’ (Male, Kanyamasinga, Farmer)

‘Let’s say you had a lot of money and then you become broke. When you become broke and you think too much, it might cause you to become mentally ill.’ (Female, Buhoma, Seamstress)

‘Some of these, there is mental illness brought by family issues. If there is disagreement within the family, then the person gets mental illness.’ (Male, Buhoma, Farmer)

‘Unemployment, or lack of what to do, this later becomes a passage of bad thoughts [*an empty mind is a devil’s workshop*] and the person starts thinking of unconstructive things like “let me go and steal from so-and-so.” To sum it all, poverty, in a way causes mental illness.’ (Male, Mukono, Farmer)

### Intrinsic causes of mental illness

Mental illness was often described as being related to inherent failures or weaknesses of an individual. Several participants described certain constitutions as being more susceptible to mental illness, such as those who think too much, who lose their tempers or those who make poor choices:

‘When a person thinks too much for example when they don’t have money and it causes mental illness.’ (Female, Buhoma, Farmer)

‘Each and every one can suffer mental illness depending on their thoughts and their makeup/how they were brought up or their character.’ (Male, Mukono, Farmer)

‘For me I think that for a person to get mental illness, they are thinking too much. When you think too much, you get mind disturbance.’ (Male, Buhoma, Transportation Provider)

‘I think that mental illness is brought about by too many thoughts; when this happens and the head gets squeezed with many thoughts, you may get head pains and at times madness is the likely result.’ (Male, Kyumbugosho, Custodian)

‘Mentally ill people are not the same. Some people make themselves mad due to smoking, using drugs, etc. because upon starting smoking marijuana, one is very normal but after adopting these, they become destructive and ruin their lives.’ (Male, Nkwenda, Community Health Worker)

‘Mental illness can also result due to too much agony, losing of temper, or too many troubles; for instance losing all your children in a short period of time can make one overstressed and ultimately cause mental illness to the person who has been befallen by this calamity.’ (Male, Mukono, Groundkeeper/Farmer)

### Treatment of mental illness

Beliefs in causes of mental illness were also observed to guide beliefs of how mental illness should be treated. Based on whether an individual’s mental illness was believed to be internally or externally driven, different treatment options, including medical, non-medical, and a combination of the two, were described as being effective. Such interventions are described as follows.

Medical interventions cited by subjects as being effective treatments for mental illness centred on the idea of the hospital being the source for treatment and even cures for psychiatric disease. Additionally, surgeons and health workers were described as being key individuals involved in treating people for their illnesses.

‘A mental patient should have rights because the person needs to eat so that’s why we say if this illness is realised in someone this person can be taken to the hospital for treatment if there are drugs that could help with this illness then becomes normal like other people.’ (Male, Kanyashande, Shopkeeper)

‘I think that at the community we would think positively when a person has got mental illness, plan how best this person can be taken to the hospital and be seen by doctors and know what has happened to their heads.’ (Male, Kyumbugosho, Farmer)

‘The only way to help these persons is to get in touch with their families, make sure that they are taken to the hospital to access treatment. That’s the core thing that we should do as a community.’ (Female, Nkwenda, Farmer)

‘From the status of BCH that we know, they should have a separate ward and get all the drugs possible that can work upon and cure patients of mental illness.’ (Male, Nkwenda, Farmer)

Certain causes of mental illness were perceived as requiring non-medical interventions that could not be provided by a hospital. Several of these treatments are focused on resolving the extrinsic and intrinsic factors believed to be contributing to a given individual’s mental illness. Such interventions include prayer, resolution of family or personal conflicts, and the use of community-based witch doctors.

‘Even when you give yourself to God, and you pray and get saved, you are likely to get healed. For sure if you pray God on your family and you settle it in togetherness, that disease is likely to get healed.’ (Female, Buhoma, Spiritual Leader)

‘If you were brought up in a God loving/respecting/fearing families or if you quickly rush to a church for prayer upon getting any mental illness and accept God as your personal savior, those problems will be history if you are born in good families that do not believe in satanic powers.’ (Female, Nkwenda, Community Member, Spiritual Leader)

‘Some of them don’t heal, especially those caused by evil spirits; when you get prayed for you get well.’ (Female, Kanyamasinga, Gorilla Keeper)

A commonly held belief among focus group participants involved the need to tailor treatment of mental illness to the underlying cause of disease by utilising a combination of medial and non-medical treatment approaches. Such treatment often involved a mix of spiritual and pharmacologic solutions:

‘I just think that mental illness that’s associated with demons/satanic powers can be treated by community based witch doctors but illness due to epilepsy has to be referred to the hospital immediately.’ (Female, Nkwenda, Farmer)

‘There are those that can heal and those that cannot, when you go to the hospital, there are tablets you are given to swallow every day and when God has mercy on you, you get well but there are those that don’t go away and you are taken for prayers and you get fine.’ (Female, Kanyamisinga, Farmer)

‘Like I had testified earlier before and I was taken to the church to be prayed for, but I did not recover completely. I realized that is until I needed to go to the hospital for adequate care, and then got to the hospital and was given treatment for mental illness. That’s what I can testify.’ (Male, Kyumbugosho, Farmer)

‘Even the health worker that is administering the medicine will seek for God’s guidance so as to make the medicine work. Also, there are times when health workers give you the medicine and advise you to on top of it pray so that it works pretty well. So they are both necessary.’ (Female, Nkwenda, Farmer)

## Discussion

There is a significant need to establish tailored mental health services.^34,35,36^ An understanding of perceptions of the aetiology and treatment of mental illness is vital in ensuring that treatment of mental illness is focused on addressing all concerns regarding that individual’s mental health.^6^ The purpose of this study was to gain an understanding of community stakeholder perceptions regarding the concepts of mental health and its treatment. Our study demonstrates gaps in perceptions of mental illness among mental health stakeholders, thus identifying the need for further education of providers and community members. These gaps, including misconceptions about the shame, weakness, and moral integrity associated with mental illness, contribute to stigma and are essential to address in the community. Thematic analysis of participant responses to discussion questions reveals a distinct dichotomy between extrinsic biological and spiritual causes and intrinsic causes from mental weakness, poor choices or bad behaviour resulting in divine retribution. These differences are also seen to guide beliefs about the proper treatment of mental illness. The results of this study support the attribution theory, which explains the relationship between stigmatising attitudes and discriminatory behaviour.^37^ For example, persons who believe that an illness is brought on by satanic powers are more likely to turn to the church or a witch doctor for help than the hospital.

Another important issue revolving around underdeveloped mental health services is the need to discard the idea that the Western approach is the only way, moving away from the Western biological approach and incorporating cultural strengths and resilience into assessment and training of local personnel. With over 43 different languages and dialects spoken in Uganda, particularly in resource-poor areas, non-specialists may provide better care than highly trained professionals because of common cultural, linguistic and social orientation. A lack of mental health personnel and access to services highlights the utility of community-based services.^38,39^ In light of the significant belief in the interaction of faith in medicine, programmes such as BCH may consider the utility of partnering with religious leaders and faith-based healers in communicating with patients and their families. The importance of such partnership has long been a part of the discussion surrounding the development of mental health programmes in Africa.^40,41^

Recent studies have compared a variety of additional models for community-based psychiatric services, noting a significant lack of health workers.^42^ In working to develop programmes to educate workers to meet this need, BCH may benefit from the utilisation of training strategies shown to be most effective in training community health workers; such strategies include regular monitoring and collection of feedback from trainees, utilising trainee feedback to tailor and improve curriculums, prioritising interactive sessions over didactic teaching and monitoring community mental health outcomes as training progresses.^43^

Our study adds to the small but growing body of literature on the status of mental health in rural sub-Saharan Africa and the perception of community-based mental healthcare.^5,9,34,38^ Mental healthcare programmes may best be designed to meet unique community needs and should focus on the reduction of stigma regarding mental illness. The present study emphasises the importance of understanding community stakeholder perspectives in the pursuit of a decentralised mental healthcare system such as that being established in the BCH catchment area.

Limitations of the study included time and resource constraints, which limited the range of respondent types and geographical coverage of the study and which may have affected the diversity of views. The findings may not be generalised; in particular, they might not reflect the views of Ugandans or Africans in other areas. As an example of qualitative research into community mental health systems, our thematic analysis is not comprehensive, and several other perceptions of mental illness may have emerged if a larger sample of individuals had participated. Additionally, in creating our sample, we combined both the randomised proportional sample and the snowball sample to make sure we would get an adequate sample of persons with mental illnesses and leaders who have disproportionate weight in determining care and punishment in mental illness. Certainly these were not ‘hidden populations’ and one of the limitations of our methodology was not interviewing these groups of stakeholders separately.

## Conclusion

In the process of developing a community-based mental healthcare programme, staff at BCH partnered with researchers to assess community perceptions of mental illness. As the results of the present study demonstrate, community perceptions of the intrinsic and extrinsic causes of mental illness, as well as beliefs about how to treat mental illness, will need to be addressed in establishing effective programmes. The present study provides BCH staff with information on community views on mental illness and will presumably form part of a series of studies conducted with BCH as part of a quality improvement project to establish more effective community-based mental healthcare that could serve as an example for other LMIC working to create their own community mental health programmes.

## Appendix 1

### Discussion Guide

#### Opening:

I would like to start by thanking everyone who has come here today. We are here to learn about what people in your community think about mental health. The information you share today will be useful to the hospital in the hopes of improving the health of the community. All of your responses are confidential and will not be shared for uses other than the Bwindi Community Hospital mental health quality assessment study. We ask that all of you keep this information confidential as well.

The focus group should take about one and a half hours. This is a group discussion so you do not need to wait to be called on, but please speak one at a time so we can get your opinions on the tape – if you can remember to say your name before you speak, this would be helpful to us.

We want to have an open discussion about your views on mental health in order to hear the voices of individuals within the community. We are interested in all your ideas, comments and suggestions. This is not a survey and there are no right or wrong answers. We want both positive and negative comments. We ask you to please respect one another and the opinions of others. Please feel free to disagree with each other, respectfully of course, and to ask the group questions.

We particularly want to thank you for agreeing to discuss this topic with us. It can be a private subject – so thank you for including us in your circle today. It always helps when discussing things that are sensitive and private if we know each other a little better, so let’s start by getting to know each other.

Let me begin with introductions. My name is ------- and I will be acting as the facilitator for the discussion today. ------ is our co-facilitator, and ----- will be taking notes of the discussion. Now, could you please share your names, ages and professions with us? Thank you so much. Let us now begin our discussion.

1. What is mental illness? ***Oburwire bwomutwe nibuha?***
  - *Prompt*: What are signs of mental illness?
    ***Okwongyerra Kubuuza: okarebera ahariki kugira ngu omanye ngu omuntu aine oburwiire bwomutwe?***
2. What causes mental illness? ***Oburwire bwomutwe niburetwaki?***
  - *Prompt:* Is mental illness a disease? ***Okwongyerra Kubuuza: okutabuka omutwe, noburwire?***
  - *Prompt:* Is it caused by: genetic inheritance? Substance abuse? Bad things happening to the person? Brain disease? Personal weakness? God’s punishment? Imbalance of neurotransmitters? Polluted air? Spirits? Witchcraft? Planets and stars?
3. What do people in your community think about people with mental illness? ***Okwongyerra Kubuuza: Abantu omukyaro kyanyu abantu nibatekateekaki aha bantu abiine oburwire bwomutwe***
  - *Prompt:* Are people with mental illness capable of working? ***Abantu abeine oburwire bwomutwe nibabasa kukora emirimo?***
  - *Prompt:* Can anyone have mental illness? ***Buri muntu weena nabaasa kugira oburwire bwomutwe***
  - Do people in your community blame the person for their condition? ***Abantu omubyaro byanyu nibetorereza ahamumuntu ahabwoburweire bwe***
  - *Prompt:* Can you tell a person has a mental illness by looking at them? ***Okabasa kumanya ngu omuntu aine oburweire bwomutwe wamureeba Kusha (kurugirira nkuko ari kushusha***
  - *Prompt:* Are people with mental illness usually dangerous? ***Abantu abaine oburwire bwomutwe noteekateeka nibakora akabi burijo***
4. How would you feel if you were diagnosed with mental illness? ***Okahurira ota, kubakukugira ngu oine oburwire bwomutwe***
  - *Prompt:* Sad? Ashamed? Hopeful? ***Nikikusaasa? Nikikwisa eshoni? Noguma namatsiko?***
5. What relationships would you have with a person with mental illness? ***Okagira kukwatanisa/enkoraganaki nomuntu oine oburwire bwomutwe***
  - *Prompt:* Would you be friends with them? ***Okaguma orimunywani waabo?***
  - *Prompt:* Would you marry someone with mental illness? ***Okatasya/oshwera omuntu oine ourwire bwomutwe?***
6. Should people with mental illness have the same rights as people who do not have mental illness? ***Notekateeka ngu abantu abeine oburwire bwomutwe basehmereire kugira obugabe bumwe nkabantu abateine oburwire bwomutwo?***
7. Who do you think has a higher risk of getting mental illness? ***Noteekateeka ngu noha ori aha kabi kamaani kokorwara oburwiire bwomutwe?***
  - *Prompt:* Who has a lower risk of getting mental illness? ***Notekateeka ngu noha oine obuzibu bukye bwokutunga oburwire bwomutwe?***
8. Do children suffer from mental illness? ***Abaana baton abo nibarwara oburwire bwomutwe?***
9. Do people in your community think mental illness is treatable? ***Abantu okyaro kyawe (omukayaro kyanyi) nibatekateeka ngu oburwire bwomutwe nibutambwa?***
  - *Prompt:* Who should treat mental illness? ***Noha oshemereire kutambira oburwire bwomutwe?***
  - *Prompt:* How should these patients be treated? How can they improve? ***Abantu abarwire omutwe bashereire kutambirwa bata? Nibabasa kuterera bata?***
10. Do most people with mental illness get better? ***Abantu abingi abeine oburwire bwomutwe nibakira kuba gye?***
11. Where should people with mental illness live? ***Abanu abeine oburwire bwomutwe bashemereire kutura nkahe?***
12. Who (if anyone) should people with mental illness tell about their condition? ***Nimuntuki (kwarabe arihio) owabantu abeine oburwire bwomutwe bashemereire kugambira?***
  - *Prompt:* Should they tell their family? Friends? ***Nogira ngu bashemereire kugambira abeka? Abanywani?***
13. What do you know about psychiatry? ***Nomanyaki ahabwokutamba oburwire bwomutwe?***
  - *Prompt:* Is psychiatry a branch of medicine? ***Notekateka ngu okutamba oburwire bwomutwe nabwe nobushaho?***
  - *Prompt:* What do psychiatrists do? ***Abashaho boburwire bwomutwe nibakoraki?***
  - *Prompt:* When would you visit a psychiatrist? ***Niryari obu oshemereire kureba omushaho woburwire bwomutwe?***
14. Do you have faith healers in your community? ***Noyikiririza omubanyandini abarikutamba omukyaro kyawe?***
  - *Prompt:* What role do they play in treating mental health*?* ***Noteekateeka ngu beine mugashoki omukutamba oburweire bwomutwe/okugira ebiteekateeko birungi?***
15. How do people with mental illness contribute to society? ***Abantu abeine oburwire bwomutwe nibongyera ki omubantu?***
  - *Prompt:* Should people with mental illness get married? ***Abantu abeine oburwire bwomutwe bashemerereire kushwera/kushwerwa?***
  - *Prompt:* Should people with mental illness have children? ***Abantu abeine oburwire bwomutwe bashemereire kuzaara abaana?***
16. If Bwindi Community Hospital wanted to help people with mental illness, what would be the best way to do this? ***Irwariro rya Bwindi Community Hospital kuryokwenda kuhwera abantu abeine oburwire bwomutwe, niburyoki oku bakukikora?***
17. This information is very good for the hospital to improve its programmes in the community. Are there any subjects, topics, or thoughts that have not been discussed which might be useful for us to talk about? ***Ebi mwatugambira nebyomuhendo ahabwirwariro kutungura proguramu zaryo omobyaro nahairwariro, Hariho ebindi bimasho ninga ebitekateeko ebitutaganira ebiri birungi kugauiraho.***

Closing ***Okukingaho***: Think about all the things we have talked about today, which of these things would be the most important thing you would want the hospital to know? ***Tekateeka aha bintu ebitwaganiraho erizooba, nibintuki omuribyo ebikuru munonga ebiwakwenzire ngu eirwariro rimanye?***

We thank you very much for sharing your ideas and opinions with us. Your ideas will be very valuable to the hospital in providing good healthcare to the people in Bwindi. ***Nitubebaza munonga ahabwokuganira neitwe nokutuha ebitekateko byanyu. Ebitekateko byanyu nebyomuhendo munonga ahabwirwariro kuhereza obuhereza bwomutindo ahabantu ba Bwindi***.
